# Baking Powders

**Published:** 1880-12

**Authors:** 


### BAKING POWDERS

Should be made from pure cream of tartar and pure bi-carbonate of soda, and combined in such exact proportions that effervescence shall be complete ; for if there is an excess of either ingredient, such excess is not only wasted, but is a detriment to the baking powder. Owing to the difficulty that families experience in procuring these articles pure, and in properly combining them, the business of manufacturing baking powders has been almost entirely given over to practical chemists, and has become one of the great industries of the country. But, unfortunately, only a few of the manufacturers use pure materials. Cream of tartar is expensive and *alum* is cheap ; hence the temptation to employ the cheaper material, regardless of the injury it causes to allwho use it.

We some time ago caused several samples of baking powders to be analyzed by Prof. Charles Stone, of the Cooper Institute. While he found two or three perfectly pure, he considered Cleveland's Superior Baking Powder, manufactured by Cleveland Brothers, of Albany, N. Y., combined in the most accurate proportions, and, therefore, the most wholesome as well as the most economical to the consumer.

Scribner's Monthly Magazine for December contains its usual variety of attractive matter. The illustrations are of the first order, as usual. This magazine has taken, and its proprietors seem determined that it shall hold, the first place among our monthlies. It is the only American serial publication which has ever, to our knowledge, attained an extensive circulation in England, a fact which speaks well for its literary and artistic excelence.

St. Nicholas for December is fully up to the standard of former numbers. There is no periodical of its class which can compare with it in its literary matter, and the illustrations are always in the highest style. It is presumed that few households are without this magazine, but if any of our readers, who have children, are not subscribers, our advice is that they lose no time in securing so important an assistant in the instruction and amusement of the young.

